# Synergistic Effects of Psychotropics Leading to Extraordinary Weight Gain

**DOI:** 10.7759/cureus.17978

**Published:** 2021-09-14

**Authors:** Allyson J Kemp, Sana E Kazi, James L Megna, Lubov V Leontieva

**Affiliations:** 1 Psychiatry and Behavioral Sciences, State University of New York Upstate Medical University, Syracuse, USA

**Keywords:** polypharmacy, atypical antipsychotic, weight gain, extreme obesity, psychotropic

## Abstract

A 22-year-old woman had significant weight gain after being on two atypical antipsychotics, an antiepileptic, and an antidepressant for 12 months, with her weight increasing from 70 kg to 160 kg, or by 90 kg, over 16 months. This case report examines the possible synergistic effects of psychotropics, particularly two atypical antipsychotics, leading to adverse side effects, particularly severe obesity, in the context of other examined pharmacological and non-pharmacologic risk factors. Psychotropic monotherapy is the advised prescribing treatment guideline. The extraordinary weight gain resulting in severe obesity in this case demonstrates just one of the many concerns for psychotropic polypharmacy from the same sub-class of psychiatric drugs leading to increased morbidity and mortality in the psychiatric population.

## Introduction

Obesity is associated with various health risks, including diabetes, hypertension, high cholesterol, cardiovascular disease, stroke, arthritis, and certain cancers [1]. The prevalence of obesity among U.S. adults was 42.4%; severe obesity rates were second highest in Hispanic adults and more common in women [1]. Obesity rates were high among females diagnosed with fetal alcohol spectrum (FAS) disorder and doubled with a diagnosis of intellectual disability (ID) [2,3]. Childhood obesity also disproportionately affected low-income populations and those exposed to adverse childhood events (ACEs), resulting in almost double increased odds of obesity [4,5]. Both severe socioeconomic deprivation and ACEs are associated with the development of enhanced inflammatory status and hypothalamic-pituitary-adrenal (HPA) axis dysfunction contributing to the development of obesity [6].

Almost all antipsychotics are associated with varied weight gain; this is more pronounced in antipsychotic naive patients and not clearly dose-dependent [7]. Atypical antipsychotics, which in this case treat mood instability, impulsivity, and micropsychosis, are associated with rapid weight gain in the first few weeks of therapy [8]. The time it takes for weight gain to plateau varies, depending on the antipsychotic, with 4-9 months with olanzapine and 2.5-6 months with risperidone [9,10]. Studies have shown that patients on risperidone had a mean weight gain of 3.9 kg at 12 weeks, whereas patients on olanzapine had a mean weight gain of 7.2 kg at 12 weeks [11]. Another study showed that individuals receiving standard doses of risperidone had a mean weight increase of 0.22 kg per month with a weight gain of 3 kg at one year, whereas individuals on olanzapine had a 0.68 kg per month weight increase with a total weight gain of 8.2 kg at one year [12]. More specific to the case, a study over a period of 28 days showed olanzapine alone and risperidone alone resulted in 3.5 kg and 1.9 kg weight gain, respectively, whereas divalproex and olanzapine resulted in 3.7 kg weight gain, and risperidone and divalproex resulted in 3.4 kg weight gain [13]. Based on a systematic review, an average weight gain of 6 kg was noted over one year with standard doses of valproic acid, with weight gain peaking at around six months of therapy [14]. Citalopram, which is used for depression and anxiety, can contribute to weight gain as well with a gain of 1-1.5 kg weight over one year [15].

The metabolic mechanism of weight gain for valproic acid is unknown compared to olanzapine, risperidone, and citalopram, all of which affect serotonin, and histamine neurotransmitters. Atypical antipsychotics and antidepressant-related weight gain is polygenic and associated with specific individual genetic variants for receptors [16,17]. CYP2D6 is the minor metabolic pathway used by olanzapine, which is metabolized mainly by CYP1A2, and the main pathway used by risperidone; risperidone is believed to have the greatest ability to inhibit the metabolism of other medications, but the risk is low at usual clinical dosing [18]. Olanzapine, however, has more powerful antagonistic effects due to a higher affinity for dopamine-2 (D1D2D3 & D4) and serotonin-2 (5-HT2A/2C, 5-HT3, and 5-HT6) receptors compared to risperidone (D2 and 5-HT2) [19]. Citalopram shares the enzyme path CYP2D6 with olanzapine and risperidone, although it is a weak CYP2D6 inhibitor, with a high affinity for both serotonin and histamine ([minimal] 5-HT1A, 5-HT2C, and H1) [20,21]. Serotonin and histamine (5-HT1B, 5-HT2A, 5-HT2C, 5-HT6, and H1) have been implicated in the control of satiety, and new research has shown changes in gut microbiome [22,23]. While the human genome is known to be 99% consistent among people, gut flora is hypothesized to vary in individuals by up to 90%, thus affecting the pharmacokinetics of antipsychotics, which change gut microbiota [24].

## Case presentation

Extraordinary weight gain was recorded in a 22-year-old woman of Puerto Rican descent diagnosed with post-traumatic stress disorder (PTSD), borderline personality disorder (BPD), polysubstance abuse (tobacco one pack per day, cannabis, synthetic cannabis binges, crack/cocaine sporadically, and alcohol sporadically), FAS with ID, and a historical diagnosis of schizophrenia at age 15. She is the eight out of 10 children born to a mother with a history of depression and obesity (weighing 109 kg). While the patient’s siblings did not have a history of obesity, several of the maternal aunts and uncles are obese. The mother abused alcohol, whereas the patient was in utero, and the family system is noted to have a history of substance use. The father left the family when she was 8, which also marked the start of the patient being sexually abused by her brother until age 15. The mother lost custody of all her children, including the patient at 10 years old, due to drug charges. Childhood was filled with running away, foster homes, residential facilities, and homelessness; she received a 10th-grade education. There was a lifetime of extremely limited support outside of the healthcare system with ongoing documented episodes of physical abuse, sexual assaults, and prostitution, up until at least age 20. The first suicide attempt came around the age of 11, with 10-20 attempts to follow afterward including self-injurious behaviors including the ingestion of foreign bodies. Her behaviors are related to distress intolerance and mood dysregulation in the context of arrested development. Illicit drug use was first charted at around age 18 including alcohol, tobacco one pack per day, cannabis, synthetic cannabis binges leading to a reported seizure, and sporadic crack cocaine use at age 20 along with an uncompleted two-week stay at an inpatient rehab facility. Despite psychotropic polypharmacy and residing in a group home, she still required inpatient hospitalizations for safety and symptom management.

**Figure 1:**
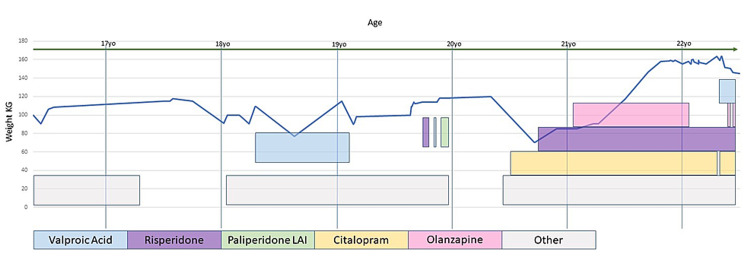
Weight and medications

The patient’s medication history is shown in Figure 1. Data had been pulled from a constellation of facilities in the New York region through the Regional Health Information Organization (RHIO) database. Medication history and weight were documented after there was a hospital visit or admission. She was hospitalized at an adolescent residential facility up until age 16 (2015). From age 18 to 22, the patient had over 50 emergency department visits and close to 40 inpatient hospitalizations. Several psychiatric medications were tried throughout her life with poor effect on symptom treatment; these include medications to treat anxiety (clonidine, guanfacine, hydroxyzine, diphenhydramine), micropsychotic features, impulsivity, and mood lability of BPD (olanzapine, risperidone, perphenazine, quetiapine, ziprasidone, paliperidone, risperidone, olanzapine, valproic acid), PTSD and depression (citalopram, sertraline), insomnia and nightmares (trazodone, prazosin, cyproheptadine, melatonin), self-injury (naltrexone), and weight gain (topiramate, metformin). She was treated for hypothyroidism (levothyroxine), although thyroid-stimulating hormone (TSH) control fluctuated outside of the institution setting due to noncompliance. Before the start of the suspect psychotropics in May 2019, the patient was not noted to be on any psychiatric medications, her weight dropped to 70 kg, and her TSH was elevated; this was the lowest documented weight for her since the age of 16. She was then maintained at a long-term inpatient psychiatric facility for seven months and discharged to a group home with her highest weight gain ever (gained 90 kg) with normal TSH. At that time, she weighed 160 kg on daily doses of olanzapine 15 mg, risperidone at 6 mg, valproic acid 1000 mg, and 20 mg citalopram for 14 months. Before this, the highest weight gain was 117 kg at 17 years of age while on both risperidone and valproic acid. After the weight gain was documented from four psychotropics, gene testing examined drug-gene interactions through GENOMIND. The testing summary is shown in Table 1 [25-32]. Citalopram had increased risk of side effects, whereas olanzapine, risperidone, and valproic acid had lower risk of side effects.

**Table 1:** Gene testing SSRI, selective serotonin reuptake inhibitor

Gene Variant	Action	Effect
Pharmacodynamic		
Serotonin transporter – SLC6A4	Low Activity	Increase side effects, such as weight gain, and increase cortisol release [25]
Brain-derived neurotrophic factor – BDNF	Altered	Influencing responses to SSRIs; recommended physical exercise to improve cognition and stress [26]
Catechol-O-methyltransferase – COMT, MET variant	Low Activity	Atypical antipsychotics produce improved responses, greater cognitive improvement [27]
5HT2C	Normal	Standard risk of weight gain [28]
Pharmacokinetic		
CYP2B6	Intermediate	Risk of elevated drug serum levels, interactions, decreased production of active metabolites [29]
UGT2B15	Intermediate	Risk of elevated drug serum levels, interactions, decreased production of active metabolites [30]
CYP1A2	Extensive	Normal activity except with inducers leading to decreased serum levels and adverse effects induced by tobacco, cannabis, coffee [31]
ABCB1	Normal	Weight gain with abnormal activity [32]

## Discussion

Psychiatric patients are often primed for weight gain even without offensive psychotropics added. Increased weight and obesity result in various health risks including metabolic syndrome associated with diabetes, hypertension, high cholesterol, cardiovascular disease, stroke, arthritis, and certain cancers. In this case, we found several biological, nonpharmacological environmental, and epigenetic factors contributing to weight gain and leading to the onset and maintenance of obesity for this patient. These factors include the patient’s Hispanic ethnicity, maternal family history of obesity, severe economic deprivation coinciding with poor diet, and surmised lack of exercise. These risk factors placed both the patient and her siblings at high risk of obesity; however, none of the patient’s nine siblings had a history of obesity, which served as a control helping to determine what exposures lead to the outcome of severe obesity for the patient alone. The patient’s family history of obesity, particularly maternal obesity and gestational weight gain, was associated with childhood obesity with this effect extending into adulthood [33]. Specific to the patient is intra-uterine exposure to alcohol leading to abnormal development as the patient is diagnosed with FAS and ID. Although the patient never had formal ACE scoring, her adverse childhood experiences, some specific to her while others are generalized to her siblings, are suspected to be high (sexual abuse by her brother, father left the family, mother’s loss of custody, placement in foster and institutions, prostitution, etc.). We do know that her high ACE scores are linked to inflammatory and neuroendocrine changes, including HPA dysfunction contributing to significant weight gain. Drug use in this case, including nicotine, alcohol, cocaine, and amphetamines, induces weight loss, whereas cannabis (data limited on synthetic cannabis) in normal-weight individuals and opiates are weight neutral [34].

From the patient’s history of weight, we can see that the patient had childhood obesity, which is known to be an independent risk factor that primed the body for further weight gain later on [35]. From age 16 to 19, the patient’s lowest weight was 70 kg and the highest was 120 kg; her weight fluctuated while on valproic acid due to noted ongoing outpatient medication noncompliance. At age 19-20, her weight again increased to 120 kg when on less than one month of non-overlapping doses of risperidone, valproic acid, and paliperidone. In her early twenties, the patient was not on psychotropics other than citalopram 20 mg for two months, and her weight dropped close to 70 kg, the lowest it had even been since age 16. After the patient was institutionalized, she was kept on citalopram 20 mg, and then three psychotropics were closely initiated together over approximately six months at standard dosing. This included risperidone at 6 mg, olanzapine 15 mg, and valproic acid 1,000 mg, less than one month apart; olanzapine and valproic acid were started less than one month apart from each other. Massive weight gain ensued at 165 kg about two months after the last medication change was made. Of importance, these four medications were administered at an inpatient facility where patients often lose weight through calorie control rather than gain weight as in this case. Prior to being on four psychotropics, the patient weighed 70 kg, with a calculated BMI of 25.7, defined to be overweight, using a height of 165.1 inches; after a weight gain of 75 kg or a bodyweight of 160 kg over 16 months, her BMI was calculated to be 58.7, defined as extreme obesity. The patient showed weight loss while taking citalopram, despite gene testing showing that the patient is at an increased risk of having increased side effects. There were no other genetic drug interactions as the patient showed the standard risk of weight gain. She stayed on risperidone for four months, which is around the time the medication weight gain is expected to plateau based on studies. Olanzapine and valproic acid were started too close to discern the effects on weight gain; however, it was not until olanzapine was stopped for four months, the antipsychotic with the highest risk of weight gain, that the patient’s weight dropped to 145 kg.

Many factors, both nonpharmacological and pharmacological, contributed to the patient's weight gain, and it is only proposed that psychotropic polytherapy, two atypical antipsychotics, caused her extreme weight gain. In this case, there is a proposed pharmacodynamic drug interaction and pharmacokinetic synergistic effect between olanzapine and risperidone, which caused extreme weight gain via the accumulation of side effects. Out of the psychotropics prescribed to the patient, olanzapine is thought to cause the most weight gain, but patients with higher baseline BMI gained significantly less weight [36]. Polytherapy is rarely evidence-based and has been shown to result in an increased risk of death, although this finding was inconsistent [37]. Combining atypical antipsychotics was associated with increased risk of metabolic syndrome, particularly with weight gain, but also had associations with reduced inpatient hospitalizations, with one case study advocating for olanzapine and risperidone polypharmacy for treatment-resistant schizophrenia [38,39]. Valproic acid, an adjunct to atypical antipsychotics, is also associated with symptom improvement in schizophrenia [13]. In this case, it is questionable if the benefits of treatment outweighed the risks of polypharmacy given the patient’s diagnoses of BPD, PTSD, and FAS with ID rather than a diagnosis of schizophrenia. Due to psychotropic polytherapy, the patient’s weight increased by an incredible 128% while also increasing the patient’s risk of mortality. There was questionable symptom management with polypharmacy as the patient continued to require inpatient hospitalizations. This case demonstrates how monotherapy has become standard practice in psychiatry. Identifying patients at risk of weight gain through careful history taking is invaluable. When patients are at higher risk of weight gain, as in this case, rather than adding medications, integration of psychotherapy skills, residential services, day treatment, and social rehabilitation will reduce the need for more medications.

## Conclusions

This case report followed a 22-year-old female diagnosed with BPD, PTSD, FAS with ID, and extreme obesity. The patient experienced 128% or 75 kg weight gain in 16 months after being started on four psychotropics, with three of the drugs started within six months, posing a significant health risk with increased mortality as the expected end result rather than symptom reduction. Despite the patient being primed for obesity from nonpharmacological factors, this case highlighted the risk practitioners take when prescribing psychotropic polypharmacy, particularly two atypical antipsychotics, risperidone and olanzapine, causing a high affinity for causing weight gain. Psychotropic monotherapy is recommended due to compounded side effects from synergistic polypharmacy, along with diligent history taking to assess which patients are at high risk of weight gain.
